# Supplementary material for: Comparative Effects of Temporal Interference and High-Definition Transcranial Direct Current Stimulation on Spontaneous Neuronal Activity in the Primary Motor Cortex: A Randomized Crossover Study
Source: Brain Sci. 2025 Mar 18;15(3):317. doi: 10.3390/brainsci15030317 (PMC11940319; doi:10.3390/brainsci15030317)
Supplement: Supplementary file 1 [file brainsci-15-00317-s001.zip › brainsci-3471620-supplementary.pdf]

S1. Blind effect and security testing

Fifteen healthy adults (male, aged 20.3 ± 1.1 years) were recruited using a double-blind crossover design. Each participant randomly received two types of stimulation: TIS ((2000 Hz, 2020 Hz, frequency difference 20 Hz), 2 mA per pair, total current: 4 mA) and HD-tDCS (total current: 2 mA). Each stimulation session lasted 6 minutes (including 30 seconds of ramp-up and ramp-down), with a 48-hour washout period between sessions. After stimulation, participants completed the subject subjective assessment scale and blinding test questionnaire. Using Pearson's chi-square test to analyze the questionnaire results, the subject subjective assessment scale revealed no significant difference in total adverse reaction scores between the two groups ($\chi2$ = 11.121, P = 0.113) (Supplemental Figure S1). Similarly, the blinding test questionnaire showed no significant difference in stimulation blinding across groups ($\chi2$ = 6.00, P = 0.107) (Supplemental Figure S2).

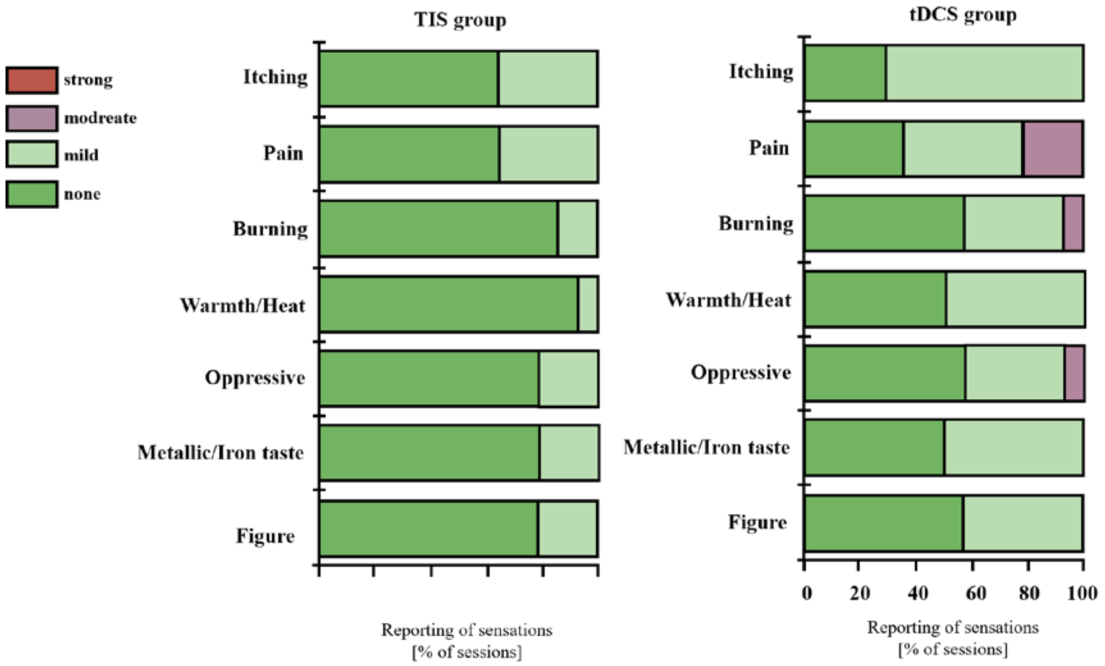

Supplemental Figure S1. Adverse reaction scores comparison between TIS group and HD-tDCS groups. Note: TIS, temporal interference stimulation; HD-tDCS, high-definition transcranial direct current stimulation.

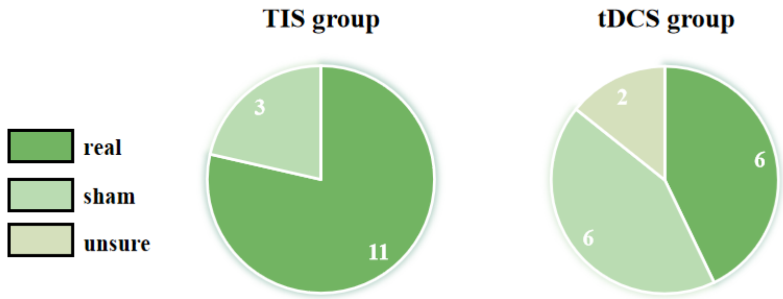

Supplemental Figure S2. Blinding efficacy assessment between TIS group and HD-tDCS groups. Note: TIS, temporal interference stimulation; HD-tDCS, high-definition transcranial direct current stimulation.

S2. Computational simulation of electrical field intensity

To elucidate the spatial electric field distribution in the human brain during transcranial electrical stimulation (TIS and tDCS), we employed finite element modeling software (COMSOL) to simulate electric field propagation. An anatomically derived head model based on the Colin27 SimNIBS template was utilized. The computational model was stratified into five discrete tissue compartments with distinct electrical conductivities: scalp (0.333 S/m), skull (0.008 S/m), cerebrospinal fluid (CSF) (1.79 S/m), gray matter (0.4 S/m), and white matter (0.15 S/m). Electrode configurations for both stimulation protocols were illustrated in Supplemental Figures S3 and S4. The spatial distribution of amplitude-modulated electric fields was quantified along the posterior-anterior axis. A quasi-static approximation of Maxwell's equations was assumed in the simulation (Huang, Y., & Parra, L. C. 2019).

"The simulated current intensity of TI and HD-tDCS was measured within a spherical region with a 10-mm radius centered at the precentral gyrus coordinates (-42, -13, 53). The results revealed that the envelope field intensity for TI was 0.541 V/m (Figure 2C), whereas the field intensity for HD-tDCS was 0.344 V/m (Figure 3C)." (P9, L27)

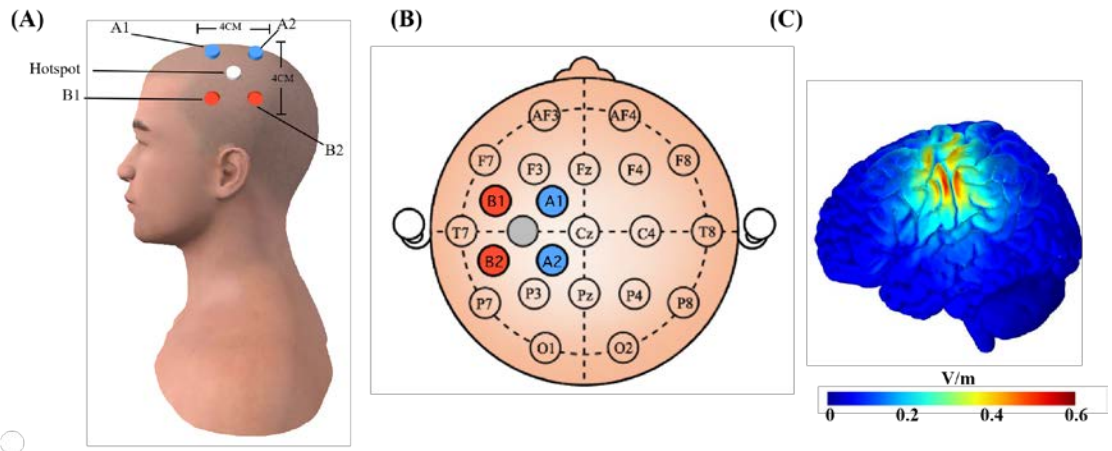

Supplemental Figure S3. Simulation model, electrode placement, and electrical field. (A) Head model with electrode placements. (B) Electrode locations based on 10-20 system. (C) Electric field simulation diagram of TI. **Note:** Stimulation Pair 1 A1-A2 (blue, 2000 Hz channel, 2 mA), Pair 2 B1-B2 (red, 2020 Hz channel, 2 mA). Grey electrode is the targeting area.

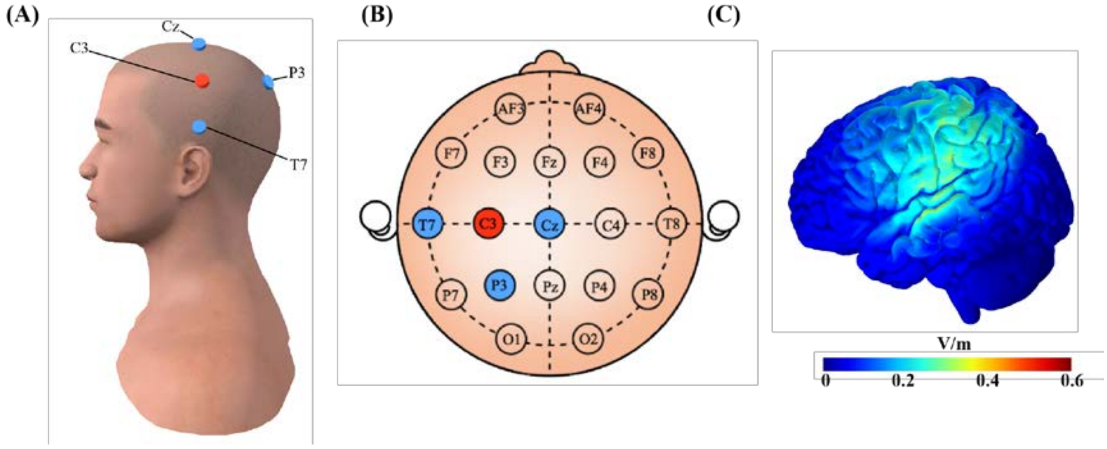

Supplemental Figure S4. Simulation model, electrode placement, and electrical field. (A) Head model with cathode (blue) and anode (red) electrodes. (B) Electrode locations based on 10-20 system: Anode (C3, 2000 μA), Cathodes (T7, -684 μA; P3, -774 μA; Cz, -542 μA). C) Electric field simulation diagram of HD-tDCS.

S3. Details of the stimulus paradigm

Temporal Interference Stimulation:

Temporal Interference Stimulation involves several key steps. First, Transcranial Magnetic Stimulation (TMS) is used for target point identification. A TMS device, set to the lowest possible stimulation intensity for safety and comfort, targets the left primary motor cortex. The target point is determined by stimulating different regions of this cortex and identifying the location that induces a visible contraction in the first dorsal interosseous muscle of the right hand, confirmed by visual inspection and EMG recordings if needed[3]. Electrode placement for Transcranial Impedance (TI) stimulation is carefully planned. Four electrodes form a 4 - cm - sided square around the target point identified through TMS. The stimulation was administered via four MRI-compatible conductive rubber electrodes, each measuring 1.5 cm × 2 cm. The square's orientation has two sides parallel to the line from the glabella to the inion and the other two parallel to the line connecting the ears, ensuring consistency and reproducibility. Electrodes are securely attached to the scalp with conductive gel and adhesive to ensure good electrical contact, and impedance between each pair is measured and recorded before stimulation. Then, the stimulation parameters for TI stimulation are set. The R1-R2 channel is set to 2000 Hz and the L1-L2 channel to 2020 Hz, based on previous research and study requirements. The envelope wave generated by these two channels has a difference frequency of 20 Hz, with an amplitude of 2 mA in each channel, modulating neural activity in the target region[4]. A Soterix Medical device from New Jersey, USA, is used for its precision and safety features. The

stimulation session protocol is established. The session lasts 20 minutes, determined by the desired effect and participant tolerability. It includes two brief 30 - second ramp-up and ramp-down phases at the beginning and end. The ramp-up phase gradually increases stimulation intensity to the target level, while the ramp-down phase gradually decreases it to zero, minimizing discomfort and adverse effects and ensuring a smooth transition into and out of stimulation.

High-Definition Transcranial Direct Current Stimulation:

The experimental protocol used an MRI-compatible DC-STIMULATOR PLUS (NeuroConn GmbH, Ilmenau, Germany) for HD-tDCS. The device settings were configured based on the parameters outlined in Esmaeilpour et al. [5]. The stimulation was administered via four MRI-compatible conductive rubber electrodes, each measuring 1.5 cm × 2 cm. Electrode placement was performed using an international 10-20 EEG system for brain localization. The STIMWEAR software simulated the stimulation montage with the left FDI-M1 as the target region, four distributed electrodes, and a total current of 2 mA. The stimulation montage obtained was as follows: 2000 μA at C3 (anode), -774 μA at P3, -684 μA at T7, and -542 μA at Cz (cathode). The total stimulation time was 20 min, which included a 1-minute ramp-up and ramp-down phase (30 s each), with real-time impedance maintained below 30 kΩ.

S4. Statistical results under different head movement exclusion criteria

Three participants were excluded from the study due to excessive head motion, with exclusion criteria set at a maximum displacement of over 3.0 mm or an angular rotation exceeding 3.0°.

Supplemental Table S1. Significant differences in ReHo.

Comparisons	Brain regions/BA	Peak MNI coordinates			Cluster Voxels	Peak t values
		X	y	z		
**tDCS S3 - tDCS S1**	Precentral_R	39	-27	60	55	4.80
	Temporal_Sup_R	54	-9	0	22	4.38
	Postcentral_L	-27	-39	66	94	4.33
	Supp_Motor_Area_R	6	-18	60	28	4.51
	Postcentral_R	30	-39	69	42	3.92
**tDCS S3 - tDCS S4**	Postcentral_R	45	-27	60	18	4.66
	Temporal_Sup_R	54	-12	3	29	4.62
**TI S2 - TI S1**	Precentral_L	-33	-30	60	79	5.30
	Postcentral_L	-51	-18	39	24	4.34
	Insula_R	48	-3	0	27	4.10
	Insula_R	36	-15	15	20	3.92
**TI S3 - TI S1**	Temporal_Sup_L	-45	-24	9	181	5.44

	Postcentral_R	30	-33	60	284	6.30
	Postcentral_L	-27	-33	60	378	5.12
	BA48_R	33	-18	12	192	5.00
	Supp_Motor_Area_R	6	-15	54	20	4.47
**TI S4 - TI S1**	Precentral_R	51	-18	45	92	5.13
	Postcentral_L	-48	-18	51	52	4.65
**TI S3 - TI S2**	Temporal_Sup_L	-48	-15	0	46	5.00
	Postcentral_R	30	-33	60	38	4.71
	Postcentral_L	-33	-42	57	22	4.29
**TI S3 - TI S4**	Postcentral_R	30	-33	60	42	5.14
	Temporal_Sup_R	60	-33	9	25	5.00
	BA48_R	33	-18	9	61	4.78
	Heschl_L	-45	-15	6	67	4.41

Notes. BA, Brodmann's area, L, left; R, right; S1: baseline; S2: first half of the stimulus; S3: second half of the stimulus; S4: post-stimulus; TI, Temporal Interference stimulation; tDCS, transcranial Direct Current Stimulation; ReHo, Regional Homogeneity; Interaction, the stimulation type× time interaction effect assessed via two-way repeated measures ANOVA. Report results with edge - level p<0.001 and cluster - level p<0.05.

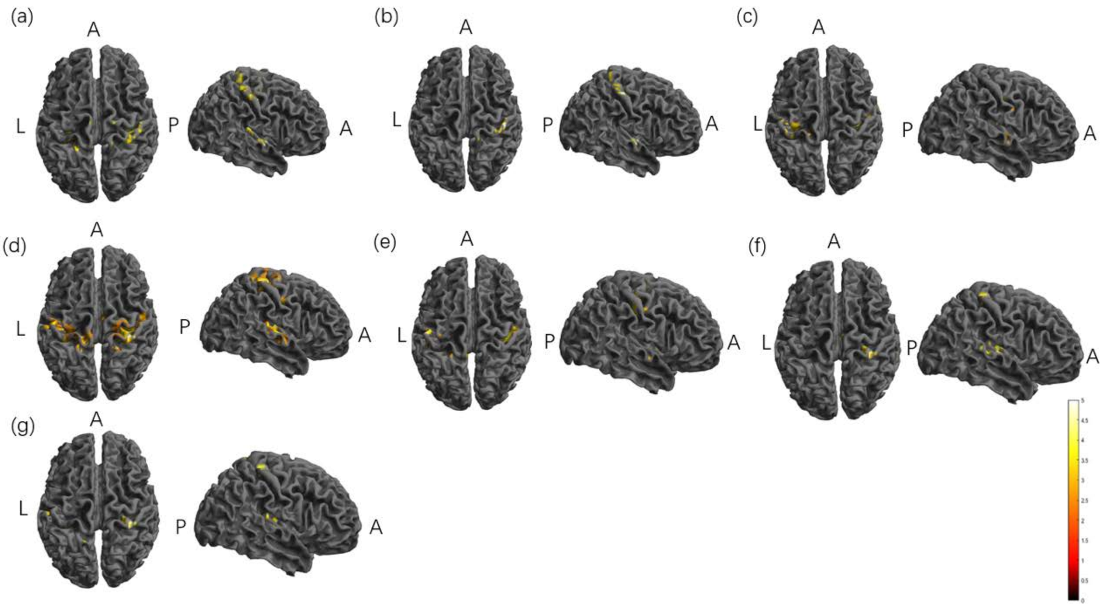

Supplemental Figure S5. The significant differences in brain regions of ReHo. (a) Between tDCS-S3 and tDCS-S1. (b) Between tDCS-S3 and tDCS-S4. (c) Between TI-S3 and TI-S1. (d) Between TI-S3 and TI-S2. (e) Between TI-S4 and TI-S1. (f) Between TI-S3 and TI-S2. (g)Between TI-S3 and TI-S4. Note: The color bar represents the T value; S1: baseline; S2: first half of the stimulus; S3: second half of

the stimulus; S4: post-stimulus; TI, Temporal Interference stimulation; tDCS, transcranial Direct Current Stimulation; A, anterior; R, right; P, posterior. ReHo, Regional Homogeneity; Report results with edge - level p<0.001 and cluster - level p<0.05.

Supplemental Table S2. Significant differences in dReHo.

Comparisons	Brain regions/BA	Peak MNI coordinates			Cluster Voxels	Peak t values
		X	y	z		
**tDCS S1 – tDCS S3**	Temporal_Sup_R	54	-18	6	29	4.90
	Postcentral_L	-33	-45	66	29	4.64
	Supp_Motor_Area_R	6	-21	57	38	4.51
	Temporal_Sup_L	-57	-21	6	37	4.50
	Precentral_R	48	-15	39	34	4.43
**tDCS S4 – tDCS S3**	Precentral_R	24	-30	72	188	5.18
	Temporal_Sup_R	57	-9	3	54	4.48
**TI S1 – TI S3**	Heschl_L	-42	-27	9	51	5.52
	Precuneus_L	-15	-45	66	45	5.18
	Postcentral_R	27	-30	60	68	5.07
	Postcentral_L	-45	-18	51	69	4.98
	Paracentral_Lobule_L	-6	-36	72	39	4.85
	Postcentral_R	12	-39	72	24	4.78
	Heschl_R	48	-18	9	67	4.60
**TI S4 – TI S3**	Temporal_Sup_R	60	-36	9	21	5.03
	Heschl_R	48	-18	9	29	4.74
	Supp_Motor_Area_R	6	-3	45	23	4.49
	Temporal_Sup_L	-42	-24	6	50	4.45
	Postcentral_R	27	-27	57	25	4.43

Notes. BA, Brodmann's area, L, left; R, right; S1: baseline; S2: first half of the stimulus; S3: second half of the stimulus; S4: post-stimulus; TI, Temporal Interference stimulation; tDCS, transcranial Direct Current Stimulation. dReHo, dynamic, Regional Homogeneity; Interaction, the stimulation type× time interaction effect assessed via two-way repeated measures ANOVA. Report results with edge - level p<0.001 and cluster - level p<0.05.

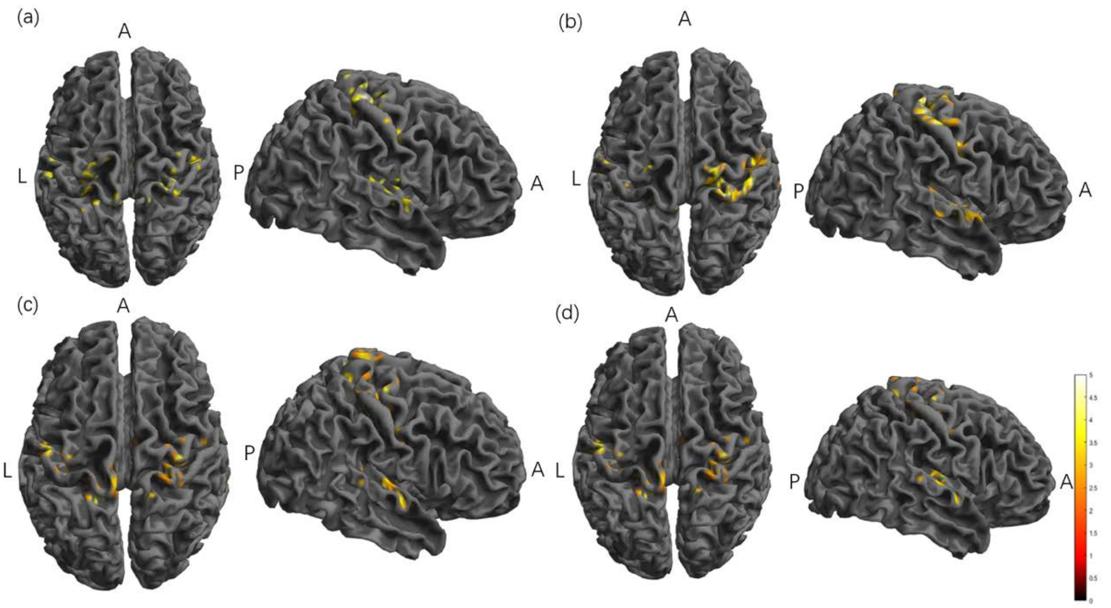

Supplemental Figure S6. The significant differences in brain regions of dReHo. (a) Between tDCS-S1 and tDCS-S3. (b) Between tDCS-S4 and tDCS-S3. (c) Between TI-S1 and TI-S3. (d) Between TI-S4 and TI-S3. Note: The color bar represents the T value. S1: baseline; S3: second half of the stimulus; S4: post-stimulus; dReHo, dynamic ReHo; TI, Temporal Interference stimulation; tDCS, transcranial Direct Current Stimulation; A, anterior; R, right; P, posterior; S, superior. Report results with edge - level $p<0.001$ and cluster - level $p<0.05$.

Reference:

1. Fabri, A.; Giezeman, G.J.; Kettner, L.; Schirra, S.; Schönherr, S. On the design of CGAL a computational geometry algorithms library. *Software: Practice and Experience* **2000**, *30*, 1167-1202.
2. Dular, P.; Geuzaine, C.; Henrotte, F.; Legros, W. A general environment for the treatment of discrete problems and its application to the finite element method. *IEEE transactions on magnetics* **1998**, *34*, 3395-3398.
3. Conforto, A.B.; Z'Graggen, W.J.; Kohl, A.S.; Rösler, K.M.; Kaelin-Lang, A. Impact of coil position and electrophysiological monitoring on determination of motor thresholds to transcranial magnetic stimulation. *Clin Neurophysiol* **2004**, *115*, 812-819, doi:10.1016/j.clinph.2003.11.010.
4. Violante, I.R.; Alania, K.; Cassarà, A.M.; Neufeld, E.; Acerbo, E.; Carron, R.; Williamson, A.; Kurtin, D.L.; Rhodes, E.; Hampshire, A.; et al. Non-invasive temporal interference electrical stimulation of the human hippocampus. *Nat Neurosci* **2023**, *26*, 1994-2004, doi:10.1038/s41593-023-01456-8.
5. Esmaeilpour, Z.; Shereen, A.D.; Ghobadi-Azbari, P.; Datta, A.; Woods, A.J.; Ironside, M.; O'Shea, J.; Kirk, U.; Bikson, M.; Ekhtiari, H. Methodology for tDCS integration with fMRI. *Hum Brain Mapp* **2020**, *41*, 1950-1967, doi:10.1002/hbm.24908.
